# A Dietary Supplementation with Leucine and Antioxidants Is Capable to Accelerate Muscle Mass Recovery after Immobilization in Adult Rats

**DOI:** 10.1371/journal.pone.0081495

**Published:** 2013-11-29

**Authors:** Isabelle Savary-Auzeloux, Hugues Magne, Carole Migné, Marion Oberli, Denis Breuillé, Magali Faure, Karine Vidal, Marie Perrot, Didier Rémond, Lydie Combaret, Dominique Dardevet

**Affiliations:** 1 Clermont Université, Université d'Auvergne, Unité de Nutrition Humaine, BP 10448, Clermont-Ferrand, France; 2 INRA, UMR 1019, UNH, CRNH Auvergne, Clermont-Ferrand, France; 3 Nestlé Research Center, Vers-chez-les-Blanc, Lausanne, Switzerland; National Institute of Agronomic Research, France

## Abstract

Prolonged inactivity induces muscle loss due to an activation of proteolysis and decreased protein synthesis; the latter is also involved in the recovery of muscle mass. The aim of the present work was to explore the evolution of muscle mass and protein metabolism during immobilization and recovery and assess the effect of a nutritional strategy for counteracting muscle loss and facilitating recovery. Adult rats (6–8 months) were subjected to unilateral hindlimb casting for 8 days (I0–I8) and then permitted to recover for 10 to 40 days (R10–R40). They were fed a Control or Experimental diet supplemented with antioxidants/polyphenols (AOX) (I0 to I8), AOX and leucine (AOX + LEU) (I8 to R15) and LEU alone (R15 to R40). Muscle mass, absolute protein synthesis rate and proteasome activities were measured in gastrocnemius muscle in casted and non-casted legs in post prandial (PP) and post absorptive (PA) states at each time point. Immobilized gastrocnemius protein content was similarly reduced (-37%) in both diets compared to the non-casted leg. Muscle mass recovery was accelerated by the AOX and LEU supplementation (+6% AOX+LEU vs. Control, P<0.05 at R40) due to a higher protein synthesis both in PA and PP states (+23% and 31% respectively, Experimental vs. Control diets, P<0.05, R40) without difference in trypsin- and chymotrypsin-like activities between diets. Thus, this nutritional supplementation accelerated the recovery of muscle mass via a stimulation of protein synthesis throughout the entire day (in the PP and PA states) and could be a promising strategy to be tested during recovery from bed rest in humans.

## Introduction

Prolonged inactivity or bed rest results in muscle wasting and in an overall loss of lean body mass (see 1 for review). Besides the obvious decreased physical performances, a reduced muscle mass impairs defences since muscle is the most important store of body amino acids (AA) useable during environmental stresses. Thus, prevention of muscle loss during the immobilization period and/or a stimulation or acceleration of muscle recovery after immobilization is important to preserve an optimum health status. This is especially important since muscle inactivity is often associated with diseases/physiological states, such as head injuries, sepsis or ageing [2,3]. Resistance exercise is highly efficient to prevent muscle protein loss during immobilization [4], however, exercise is not always relevant in specific physio-pathological situations such as invalidating diseases, joint pain or in frail elderlies. Thus, a main clinical issue in such catabolic situations is the development of new approaches to limit muscle atrophy and/or improve subsequent recovery. These new strategies could also be considered together with exercise when this later is feasible. 

The loss of muscle mass during disuse is the result of an imbalance between muscle protein synthesis and breakdown [1,5]. During immobilization, an increased activation of proteolytic systems has been well described in animals and humans (involvement of ATP-Ubiquitin proteasome pathway [6-9], calpain system [6,10], lysosomal pathway [6]) associated with a decreased protein synthesis in skeletal muscles [11-13]. The generation of an oxidative stress with an increased production of reactive oxygen species (ROS) during the immobilization period has been suspected to be partially responsible of these muscle protein metabolism alterations in both humans [14,15] and animals [16-19]. The prevention of ROS production by mitochondrial-targeted molecules or dietary antioxidants has been proven efficient in some studies to preserve muscle mass during the immobilization period [16] or at the beginning of the recovery period [20] via a reduction of proteases activities and an inhibition of apoptosis. The presence of an inflammatory state during immobilization [19] and an associated resistance of muscle metabolism to anabolic stimuli such as insulin [21] has also been clearly stated.

The experimental model of immobilization also directly induces a resistance of muscle protein synthesis to anabolic stimuli such as food intake and more precisely dietary proteins and AA [22-24]. Because proteins and AAs are robust stimulators of protein synthesis, AAs supplementation has been tested to increase muscle anabolism during prolonged immobilization. A particular attention was given to the branched chain AA (BCAA) (leucine + valine + isoleucine) or more precisely to leucine alone, which is known to stimulate muscle protein anabolism. In bed rest studies, BCAA, leucine alone or protein supplementation led to conflicting results with no impact [25] or a positive effect [26,27] on protein synthesis and muscle function. These results suggested that depending on the degree of the muscle anabolic resistance to AA during the immobilization period, leucine or AA supplementations may be inefficient to prevent muscle mass loss. Indeed, in humans, immobilization periods are often associated with pathology/physiological states linked with an increase of stress mediators (such as glucocorticoids [28]). Rieu et al (2004) have shown that exogenous glucocorticoids induced a total resistance of muscle protein synthesis to the anabolic effect of leucine which rendered skeletal muscle response insensitive to amino acids even at very high plasma concentration. In addition, it has been shown that increased oxidative stress also reduced the ability of amino acids (and leucine in particular) to stimulate muscle protein synthesis [29]. 

To allow muscle recovery, it is necessary not only to normalize the muscle protein synthesis / proteolysis ratio but also to be in a situation of a positive nitrogen balance. It is now well established that skeletal muscle proteolysis is normalized very rapidly during the first days of reloading [6,7,19]. Then, a stimulation of protein synthesis during the recovery period is a major determinant of the muscle mass recovery. Because sensitivity of muscle protein synthesis to anabolic stimuli should progressively recover during reloading, leucine supplementation may efficiently favor muscle anabolism during this period. To our knowledge, and except for [25], free leucine supplementation alone has not been tested yet during the recovery period to accelerate muscle recovery in adults.

The aims of the present study are to assess the beneficial effects of a sequential nutritional strategy to counteract muscle loss and sustain/stimulate muscle protein anabolism during immobilization and subsequent recovery in a rat model. The sequential nutritional intervention consists in a dietary supplementation of a mixture of antioxidants during the immobilization period and the first phase of reloading (i.e., when an oxidative stress occurs and proteolysis stimulated) combined with a supplementation of leucine during the recovery period (i.e., when protein synthesis stimulation is essential and most efficient for an optimal protein anabolism). To our knowledge, no study has tested the combined and sequential beneficial impact of a supplementation of leucine and antioxidants on muscle protein mass and synthesis rates in immobilized adult rats.

## Materials and Methods

### Animals and experimental design

 This study was conducted in accordance with institutional guidelines on animal experimentation in France and validated by the Ethics Committee in Animal Experiment CEMEAAuvergne (registration number: CE4-09). Male Wistar rats aged 6-8 months were housed individually under controlled environmental conditions (room temperature 22°C; 12 h light-dark cycle, light period starting at 08:00 AM), fed *ad libitum* a standard 13% casein diet (Table 1) and given free access to water.

**Table 1:** Composition of the standard and the experimental diets.

	Control diet		Experimental diet		
	Casein standard diet	ALA	AOX	AOX+LEU	LEU
Ingredients (g/kg dry matter)					
Casein	166	166	166	166	166
L cystine	1.8	1.8	1.8	1.8	1.8
Alanine^a^	-	59	-	-	-
Leucine	-	-	-	44.5	44.5
Valine^b^	-	-	-	5.1	5.1
Isoleucine^b^	-	-	-	9.8	9.8
Hesperetine 7 glucoside	-	-	1	1	-
Curcumin	-	-	1.43	1.43	-
Green tea catechins	-	-	2	2	-
Rutin	-	-	2	2	-
Rapeseed oil	30	30	30	30	30
Sunflower oil	3	3	3	3	3
Peanut oil	27	27	27	27	27
Cellulose	35	35	28.57	28.57	35
Saccharose	100	100	100	100	100
Lactose	134	134	134	134	134
Wheat flour	458.2	399.2	458.2	398.8	398.8
Mineral mixture Control	35	35	-	-	35
Vitamin mixture Control	10	10	-	-	10
Mineral mixture supplemented	-	-	35	35	-
Vitamin mixture supplemented	-	-	10	10	-
Given during	Adaptation^(1)^ immobilization^2^	Recovery	immobilization	Recovery I8-R15	Recovery R15-R40
Given to	All rats 1 Pair fed rats 2	Pair fed rats	Casted rats	Casted rats	Casted rats

Diets were provided by INRA (Unité de Préparation des Aliments Expérimentaux, Domaine de Vilvert, Jouy-en-Josas, France)

Vitamin mix control expressed (/kg mix) : Nicotinic acid 3g, D-Pantothenate Ca 1.6 g, Pyridoxine HCl 0.7g, Thiamin HCl 0.6, Riboflavin 0.6 g, Folic acid 0.2 g, D-Biotin 0.02 g, Vitamin B12 (0.1%) 2.5 g, Vitamin K 0.075 g, Choline (chlorhydrate, bitartrate) 250 g, Vitamin E 1000 IU, Vitamin A 400000 IU, Vitamin D3 100000 IU.

Vitamin mix supplemented: similar to vitamin mixture control except for Vitamin E: 30000 IU and Vitamin A: 800000 IU

Mineral mix control expressed in (mg/kg mix): Calcium carbonate 357, Potassium phosphate, monobasic 250, Sodium chloride 74, Potassium sulfate 46.6, Potassium citrate monohydrate 28, Magnesium oxide 24, Ferric citrate 6.06, Zinc carbonate 1.65, Manganous carbonate 0.63, Curpic carbonate 0.3, Potassium iodate 0.01, Sodium selenate anhydrous 0.01025, Ammonium paramolybdate, 4 hydrate 0.00795, Sodium meta-silicate, 9 hydrate 1.45, Chromium potassium sulfate, 12 hydrate 0.275, Boric acid 0.0815, Sodium fluoride 0.0635, Nickel carbonate 0.0318, Lithium chloride 0.0174, Ammonium vanadate 0.0066.

Mineral mix Supplemented: similar to Mineral mix control except for Zinc carbonate 2.73 and Sodium selenate anhydrous 0.1429.

The standard diet was used for the adaptation period for all rats (1) and for the immobilization period in pair fed rats only (2)

^a^ Alanine was included in the +ALA diet to render the diets isonitrogenous. This amino acid has no effect on muscle protein metabolism; ^b^ Valine and isoleucine were included in the +AOX+LEU and the +LEU diet to prevent the fall of their plasma concentrations induced by leucine supplementation.

**Figure 1:**
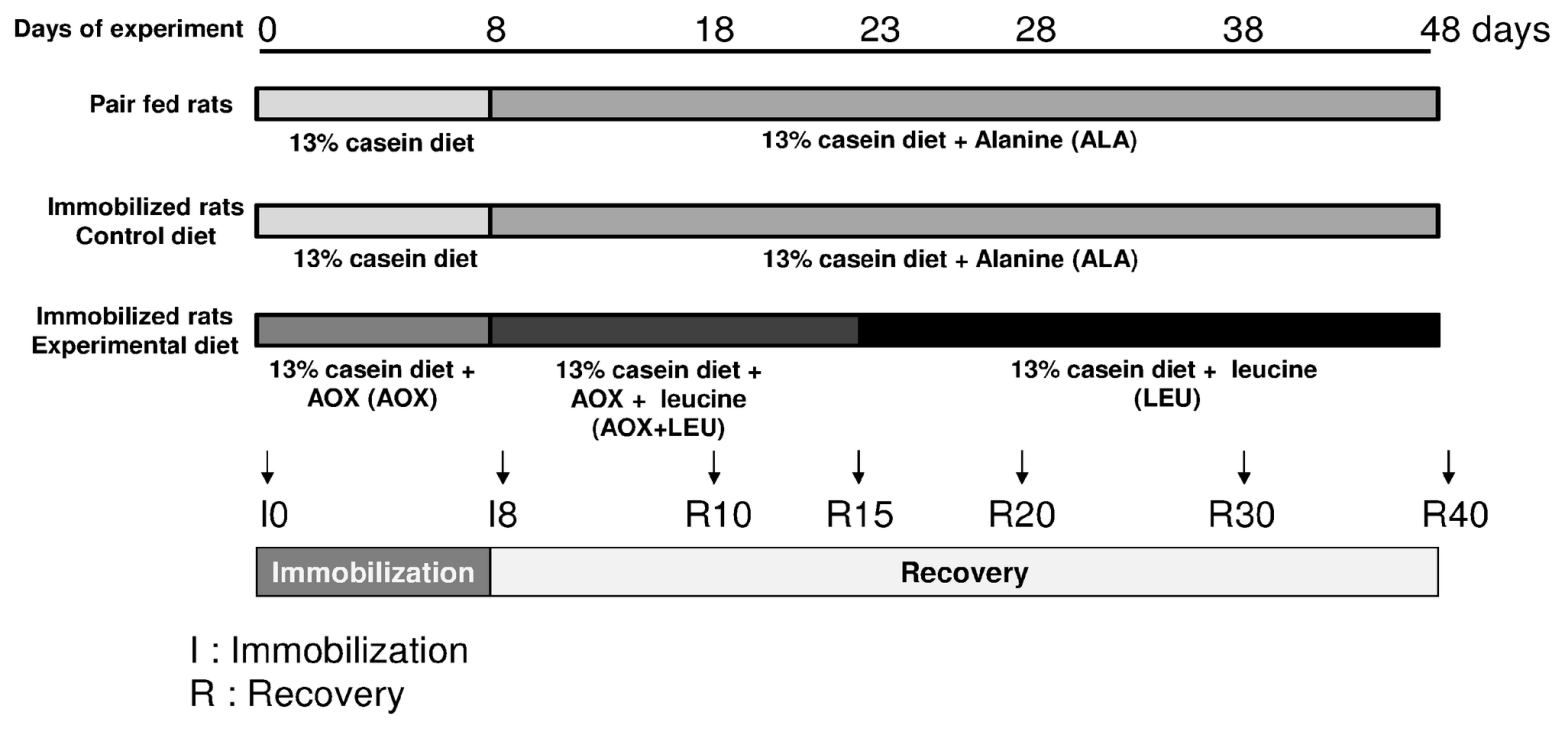
Study design used in the three groups of pair fed rats, immobilized control rats and immobilized antioxidants and/or leucine supplemented rats.

 After a 3-week adaptation period with a standard diet (Table 1), 265 rats were anesthetized with isoflurane inhalation and subjected to unilateral hindlimb cast immobilization with an Orfit-soft plaque (Gibaud, France) for 8 days (I8). The foot was positioned in plantar extension to induce maximal atrophy of the *gastrocnemius* muscle [7,19,30,31]. On day I8, casts were removed and animals were allowed to recover for 10 (R10), 15 (R15), 20 (R20), 30 (R30) or 40 (R40) days. The immobilized animals were split in 2 groups and fed one of the two experimental diets (Table 1, Figure 1). 133 immobilized rats (EXPERIMENTAL group) were fed the standard 13% casein + antioxidant (AOX) during immobilization, the diet was then switched to a 13% casein + antioxidants + 4.45% leucine (AOX+LEU) diet for the first 15 days of recovery (Figure 1) and switched to a 13% casein + 4.45% leucine diet (LEU) for the rest of the recovery period (from R15 to R40). The other 132 animals were fed a 13% casein diet (standard diet) during immobilization and the same diet supplemented with alanine (ALA) during the recovery; they constituted the control diet group (Figure 1). The LEU and AOX+LEU diets were supplemented with leucine to increase plasma leucine concentration. the LEU and AOX+LEU diets were also supplemented with appropriate amounts of valine and isoleucine to prevent the fall in their concentration induced by leucine supplementation [32]. Alanine, an amino acid which has no effect on muscle protein metabolism, was included in the standard diet (ALA) to normalize the quantity of AA given to the animals during the recovery period in the control diet group.

 Casted rats reduced their food intake during the immobilization period (from 22 g to 15 grams). Therefore, another group of 120 control non-casted rats were pair-fed to the casted rats and were fed the same diets as the ones used for the control diet group (i.e. standard diet during immobilization and ALA diet during the recovery period) (Table 1, Figure 1). 24 rats were studied as a reference point before the immobilization period (I0).

 Before immobilization (I0) and at the end of the immobilization period (I8) or at different time points during the recovery period (R10, R15, R20, R30, R40) (n=16 /diet/time point), animals were euthanized under pentobarbital sodium anaesthesia (50 mg/kg ip). The evening of the day before each time point studied, and for each diet, the food of half of the rats in each group was withdrawn, so that the rats were in a post absorptive state (PA) on the day of experiment. On the same day, the other half was fed 6 grams of their respective diet and ate all their given food in the following hour; these animals were then in the postprandial state (PP). Non-casted pair-fed rats were also studied at the PA and the PP states using the same procedure.

### Measurements of in vivo protein synthesis

 Protein synthesis rates were measured using the flooding-dose method as previously described [33]. Each rat was injected intravenously with [1-^13^C] valine (99%) (150 µmoles/100 g body), 40 min before euthanasia to flood the precursor pool with [1-^13^C] valine. Rats were then euthanized under pentobarbital sodium anaesthesia (50 mg/kg ip). Blood was withdrawn from the aorta, and hindlimb gastrocnemius and *tibialis anterior* muscles were carefully dissected, weighed and frozen in liquid nitrogen. *Gastrocnemius* muscle was chosen for protein synthesis assay because of its fibre type composition (mixed) and its size allowing multiple assays once milled. 

 Free and bound valine enrichments were determined as follows. Muscles were powdered in liquid nitrogen in a ball mill (Dangoumeau, Prolabo, Paris, France). A 200 mg-aliquot of frozen muscle powder was homogenized in 2 mL of 10% trichloroacetic acid (TCA). Homogenates were centrifuged (8000 rpm, 15 min, 4°C) and supernatants, containing free amino acids, were desalted by cation-exchange chromotography (AG 50 x 8, 100–200 mesh, H+ form, Bio-Rad, Richmond, CA) in minidisposal columns. Valine and other amino acids were eluted with 4 mol/L NH_4_OH. After evaporation of NH_4_OH under vacuum, free amino acids were resuspended in 0.01 mol/L HCl for enrichment measurements. TCA-insoluble materials were washed in 4 volumes of cold 10% TCA and 3 times in 4 volumes of 0.2 mol/L perchloric acid (PCA). Resultant pellets were resuspended in 0.3 mol/L NaOH and incubated at 37°C for 1 h. Protein concentration was determined using the bicinchoninic procedure [34]. Proteins were precipitated with 20% PCA overnight at 4°C, samples centrifuged (10,000 x *g*, 5 min, 4°C). The protein pellet was hydrolyzed in 6 mol/L HCl at 110°C for 24 h. HCl was removed by evaporation and amino acids purified by cation-exchange chromotography as described above. Measurement of free valine enrichment was done as its *t*-butyldimethylsilyl derivative by gas chromatography electron impact mass spectrometry, using a gas chromatograph coupled to an organic mass spectrometer quadrupole (GC-MS Hewlett-Packard 5971A, Hewlett-Packard Co., Palo Alto, CC=A, USA). Enrichment of [1-^13^C] valine into muscle proteins was measured as its *N*-acetyl-propyl derivatives by gas chromatography–combustion-isotope ratio mass spectrometry (Isoprime, Cheadle, UK). 

### Calculations

 The absolute synthesis rate (ASR) was calculated from the product of the protein fractional synthesis rate (FSR) and the protein content of the tissue and expressed in mg/d. FSR (in %/d) is calculated from the formula : FSR = Sb x 100/Sa x *t*, were S*b* is the enrichment at time t (minus natural basal enrichment of protein from gastrocnemius taken from rats not submitted to labelled valine injection) of the protein-bound valine, *t* is the incorporation time in d, and S*a* is the mean enrichment of free tissues valine between time 0 and *t* according to [33]. The mean S*a* enrichment was the S*a* (t_1/2_) value calculated from the linear regression obtained in tissue between time 0 and time *t*.

### Plasma amino acid measurements

Plasma amino acid concentrations were determined for each group at selected time-points I0, I8, R15 and R40. Plasma amino acids were purified, i.e., 500 µL of plasma was added to 125 µL of sulfosalicylic acid solution (1 mol/L in ethanol with 0.5 mol/L thiodiglycol) previously completely evaporated. Norleucine was added as an internal standard. Amino acid concentrations were determined using an automated amino acid analyzer with BTC 2410 resin (Biotronic LC 3000, Roucaire, Velizy, France).

### Inflammatory and oxidative markers in plasma and gastrocnemius muscle

To assess chronic inflammation, fibrinogen concentration in plasma was assessed, as previously described [35]. 

Inflammatory status in the gastrocnemius muscle was evaluated by measuring muscle content of MCP-1, a cytokine recruiting inflammatory cells. MCP-1 was detected using an ELISA kit (RayBio Rat MCP-1 ELISA RayBiotech, Inc., Norcross, GA, USA) which uses an antibody specific for rat MCP-1. The results were expressed as nanograms of MCP-1 per milligram of muscle. Oxidative status in the muscle was evaluated by measuring gastrocnemius muscle content of the total glutathione as previously described [36]. Results were expressed as µmol per gram of gastrocnemius.

### Measurement of chymotrypsin- and trypsin-like activities of the proteasomes

Samples of *gastrocnemius* muscle powder (see above “Measurements of in vivo protein synthesis”) were homogenized in 10 vol of an ice-cold buffer (pH 7.5) [50 mmol/L Tris-HCl, 250 mmol/L sucrose, 10 mmol/L ATP, 5 mmol/L MgCl_2_, 1 mmol/L DTT, and proteinase inhibitors (10 µg/mL antipain, 10 µg/mL leupeptin, 10 µg/mL pepstatin, 10 µg/mL aprotinin, and 0.2 mmol/L PMSF)] as previously described (69,70). Briefly, extracts were centrifuged at 10,000 x g for 20 min at 4°C. Supernatants were then centrifuged at 100,000 x g for 1 h at 4°C. The resulting supernatants were finally centrifuged at 100,000 x g for 5 h at 4°C. The resulting protein pellets were resuspended in 150 µL of a buffer containing glycerol (20%), 50 mmol/L MgCl_2_, and 5 mmol/L Tris·Cl, pH 7.5 (buffer B). Protein concentration was determined on these resuspended pellets using the Bio-Rad protein assay kit. The proteasome chymotrypsin- and trypsin-like activities were determined by measuring the hydrolysis of the fluorogenic substrates succinyl-Leu-Leu-Val-Tyr-7-amido-4-methylcoumarin (LLVY-AMC), and succinyl-Leu-Arg-Arg-AMC (LRR-AMC) (Enzo Life Sciences), respectively. Five microliters (~15 µg proteins) of the resuspended pellets were diluted in 15 µL of buffer B and added to 60 µL of a reaction buffer [50 mmol/L Tris·Cl (pH 8), 11.25 mmol/L MgCl_2_, 1.25 mmol/L DTT, and 0.01 U apyrase] containing either 300 µmol/L LLVYAMC or 800 µmol/L LRR-AMC to measure the proteasome chymotrypsin-or trypsin-like activities, respectively. Pilot experiments were performed with or without the proteasome inhibitor (MG132, Enzo Life Sciences) at 40 or 100 µmol/L to ensure full inhibition of chymotrypsin- or trypsin-like activities respectively. Activities were determined by measuring the accumulation of the fluorogenic cleavage product (AMC) using a luminescence spectrometer FLX800 (Biotek) for 45 min at 380-nm excitation wavelength and 440-nm emission wavelength. Then, the activities were determined by calculating the difference between arbitrary fluorescence units recorded with or without MG132 in the reaction medium. The final data were corrected by the amount of protein. The time course for the accumulation of AMC after hydrolysis of the substrate was analyzed by linear regression to calculate activities, i.e., the slopes of best fit of accumulation AMC vs. time.

### Statistical analysis

The statistical analysis was done using a non-parametric approach. Exact Wilcoxon Rank-Sum tests have been performed for all the comparisons of interest using the software R 2.14.1.

Significance was defined for P<0.05 level, tendency was defined for P<0.1.

The Hodges Lehman estimator related to the Wilcoxon test (which estimates the difference between the medians of the two groups to be compared) was used to express all the percentages of increase or decrease of one group relatively to another group (Hodges Lehman estimate divided by the median of the relative group [37]). 

Each figure is a descriptive representation of the data, using these two statistical indicators: Median and Standard Error of the Median (SE-Median)

## Results

### Food intake and body weight changes

**Figure 2:**
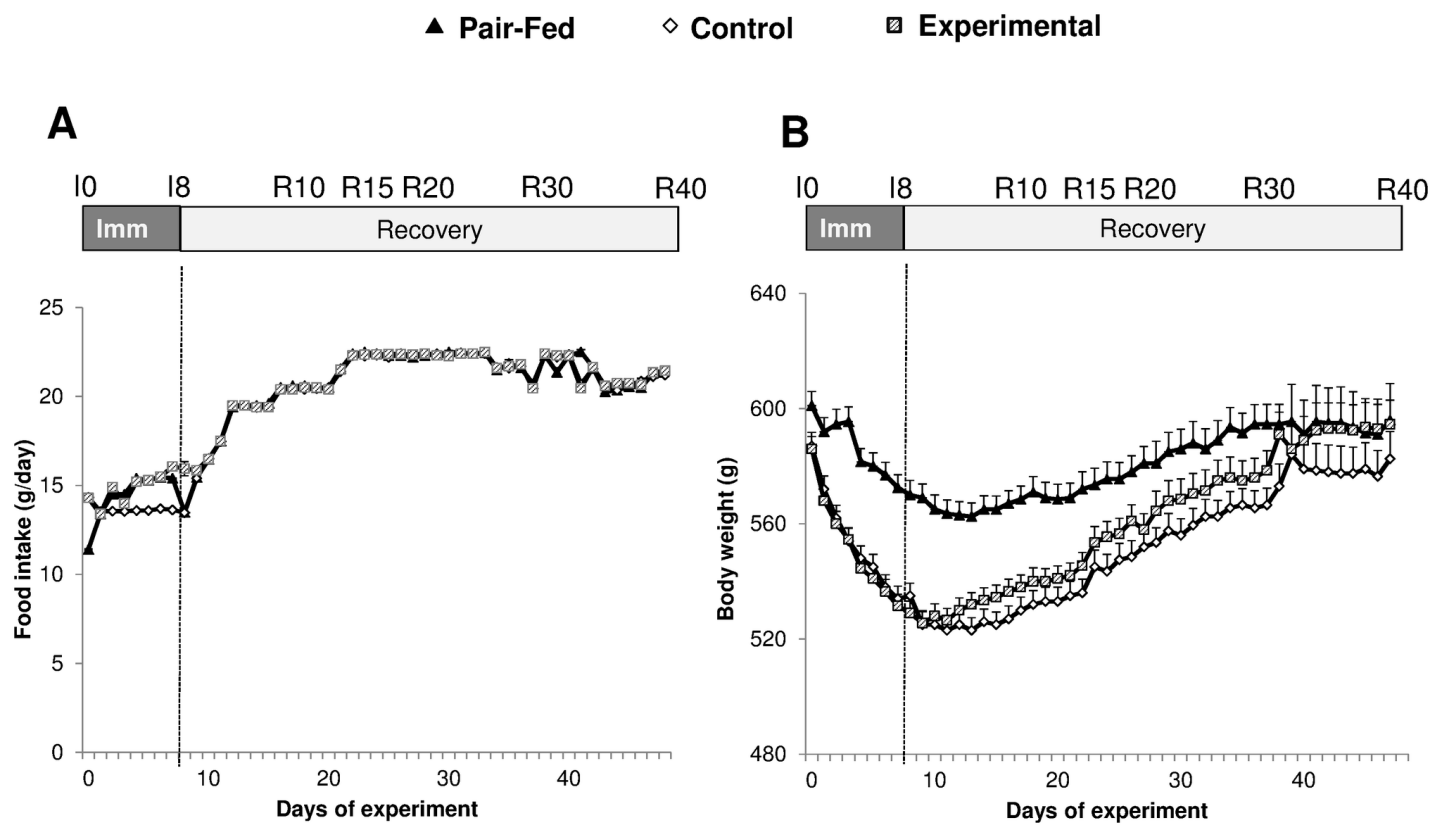
Food intake (A) (g/day) and body weight (B) (g) of casted control, casted supplemented and non-casted pair fed rats. Values are median ± SE-Median.

Before immobilization, food intake averaged 22g for all animals. Immobilization led to a lower food intake in both CONTROL and EXPERIMENTAL groups (13.5 ± 0.0 and 15.9 ± 0.4 g, respectively, Figure 2A). Food intake progressively increased throughout the recovery and reached a plateau around 20-22 g from R15 onwards in both groups. 

Body weight was reduced by 4.8% (regarding the median of all the individual reductions) in the pair fed group and by 8.9% and 9.4% in the two different casted groups at the end of the immobilization period (Figure 2B). The animals’ weight progressively increased in all groups during the recovery period (Figure 2B) to reach the following values: 582.5±9.5, 594.5±8.5 and 596±12.7 for CONTROL, EXPERIMENTAL and pair fed groups respectively (Figure 2) at the end of the recovery period. 

### Plasma amino acid concentrations

Plasma levels in leucine were not affected by immobilization in all groups (I8 vs I0, Table 2). During the recovery period, leucine is added in the in the EXPERIMENTAL diet of immobilized animals (Table 1), explaining the increased plasma concentration in the EXPERIMENTAL animals at R15 and R40 (Table 2). Concomitantly, other BCAA were also supplemented to avoid any decreased concentration in plasma due to leucine supplementation which could have limited protein synthesis. This can be shown by plasma isoleucine concentrations where no decreased concentration was observed in EXPERIMENTAL animals (Table 2).

**Table 2:** Plasma leucine, isoleucine and alanine concentrations in the post prandial state of rats.

	I0	I8			R15			R40		
		Pair Fed	Immobilized Control	Immobilized Supplemented	Pair Fed	Immobilized Control	Immobilized Supplemented	Pair Fed	Immobilized Control	Immobilized Supplemented
Leucine	106±6	116±13	104±9	94±7	106±15	115±12	286±46*	105±7	118±10	251±19*
Isoleucine	54±4	65±7	56±6	51±5	58±8	70±7	61±13	53±4	62±4	56±5
Alanine	432±19	547±84	408±31	388±21	609±79	607±57	513±19†	502±69	555±22	455±38†

Amino acid concentrations are presented at the post prandial state before (I0) and after (I8) the immobilization period but also during the recovery period (R15 and R40; 15 and 40 days of recovery, i.e. after cast removal). Values expressed in µmol/L are median ± SE-Median. * P<0.05 † P<0.1 Immobilized Control vs Immobilized Supplemented.

 In order to normalize the AA supply, alanine was supplemented in Control immobilized and pair fed animals, inducing a slight increase (+18.8%, at R40, P<0.1) of plasma alanine concentration observed in control immobilized animals relatively to immobilized supplemented (Table 2).

### Muscle mass during immobilization and recovery

**Figure 3:**
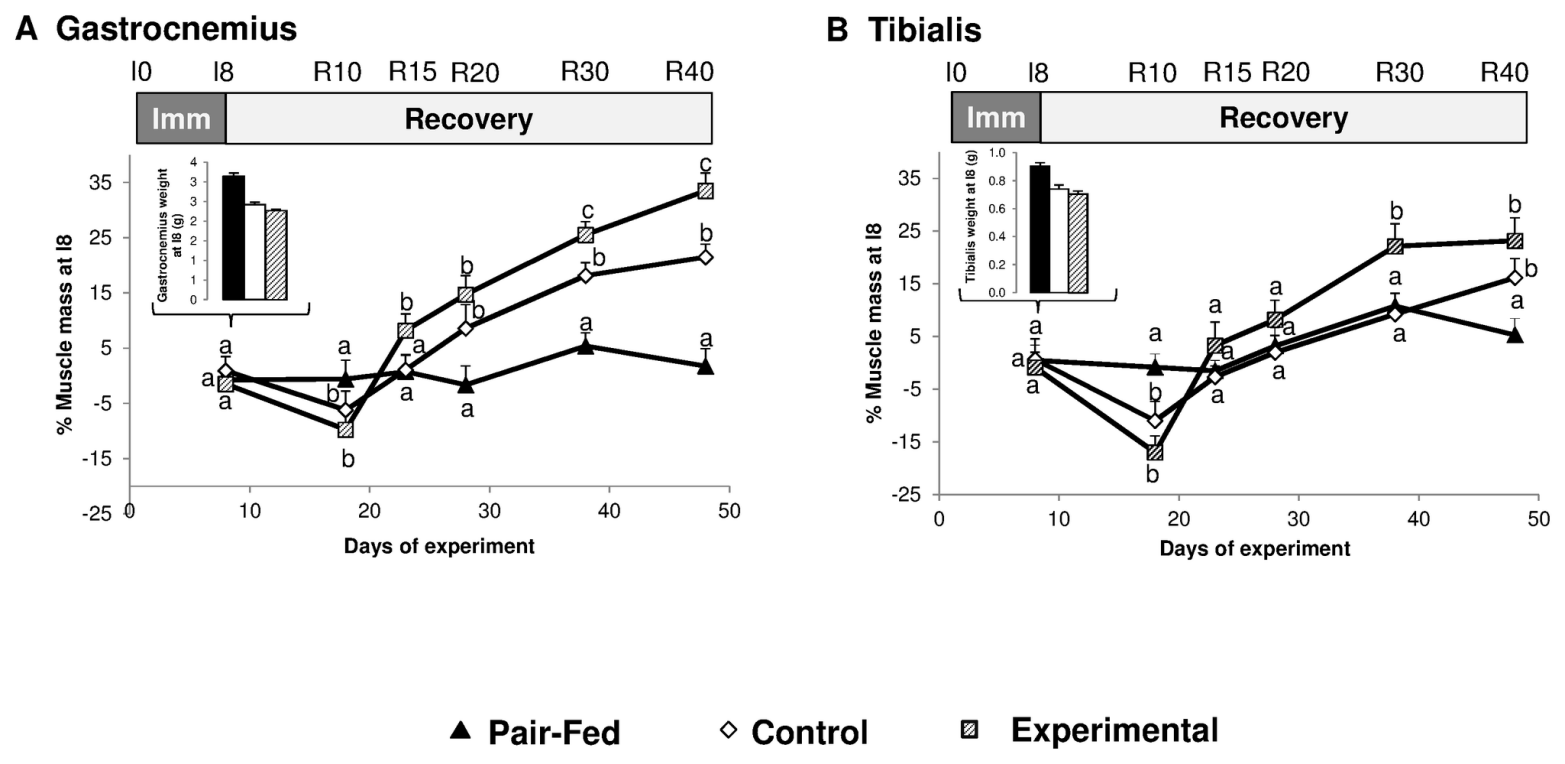
Muscle mass of the immobilized leg for casted control and casted supplemented rats and of one of the legs of the non-casted pair fed rats. The muscle mass in the 3 groups is presented at I8. The muscle mass during the recovery period is expressed as a percent (%) of the weight of the muscle at the end of the immobilization period (ie I8 time point). A: Gastrocnemius, B: Tibialis Anterior. Values are median ± SE-Median. a,b,c significantly different (non-casted Pair-fed vs casted Control vs casted AOX± Leu) (P<0.05) within each time point.

**Figure 4:**
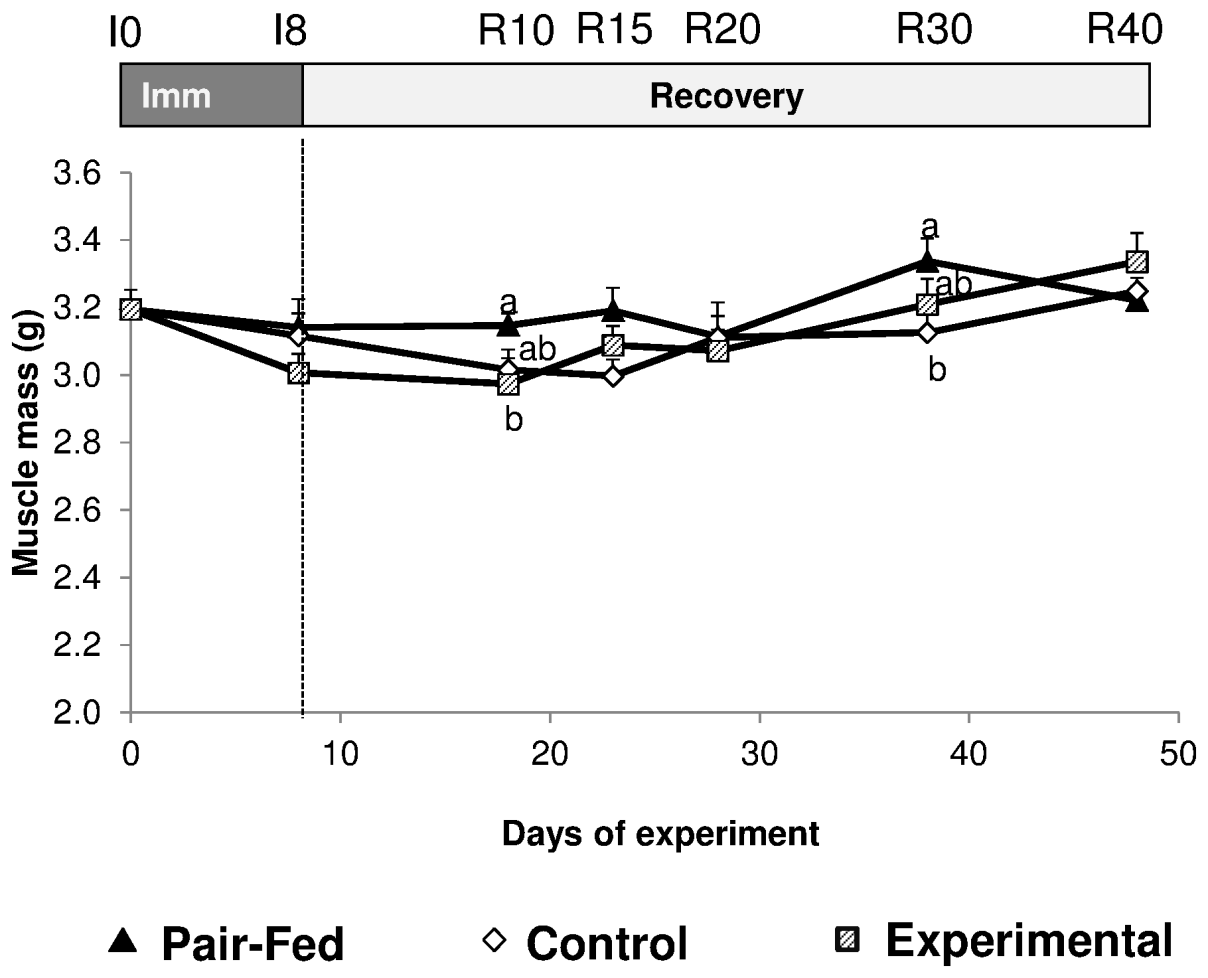
Gastrocnemius muscle mass (g) of the non-casted leg of the immobilized control and supplemented rats and of one of the legs of the non-casted pair fed rats. Values are median ± SE-Median. a,b,c significantly different (non-casted Pair-fed vs casted Control vs casted AOX± Leu) (P<0.05) within each time point.

 Immobilization induces a decrease in muscle mass (-32 and -24%, between the immobilized and the pair fed muscle at I8 for Gastrocnemius and Tibialis anterior respectively) (Figure 3). No difference between the EXPERIMENTAL vs CONTROL groups on muscle mass could be recorded at the end of the immobilization period (Figure 3). When expressed as a percentage of the muscle mass at I8, muscle mass in all immobilized muscles progressively increases during the recovery period in all muscles in EXPERIMENTAL and CONTROL relatively to the muscles of pair fed animals which were not previously immobilized (Figure 3). The percentage of recovery of muscle mass of the Gastrocnemius and Tibialis anterior muscles from the EXPERIMENTAL group was significantly (P<0.05) above the values from the CONTROL group at R30 and R40, suggesting an accelerated recovery of the muscle mass in the EXPERIMENTAL relatively to the CONTROL group. Concerning the Gastrocnemius mass in the non- immobilized leg (which could be also considered as an internal reference value), no important difference between the CONTROL and EXPERIMENTAL groups and the pair fed group was observed (Figure 4) except for small differences at R10 and R30.

**Figure 5:**
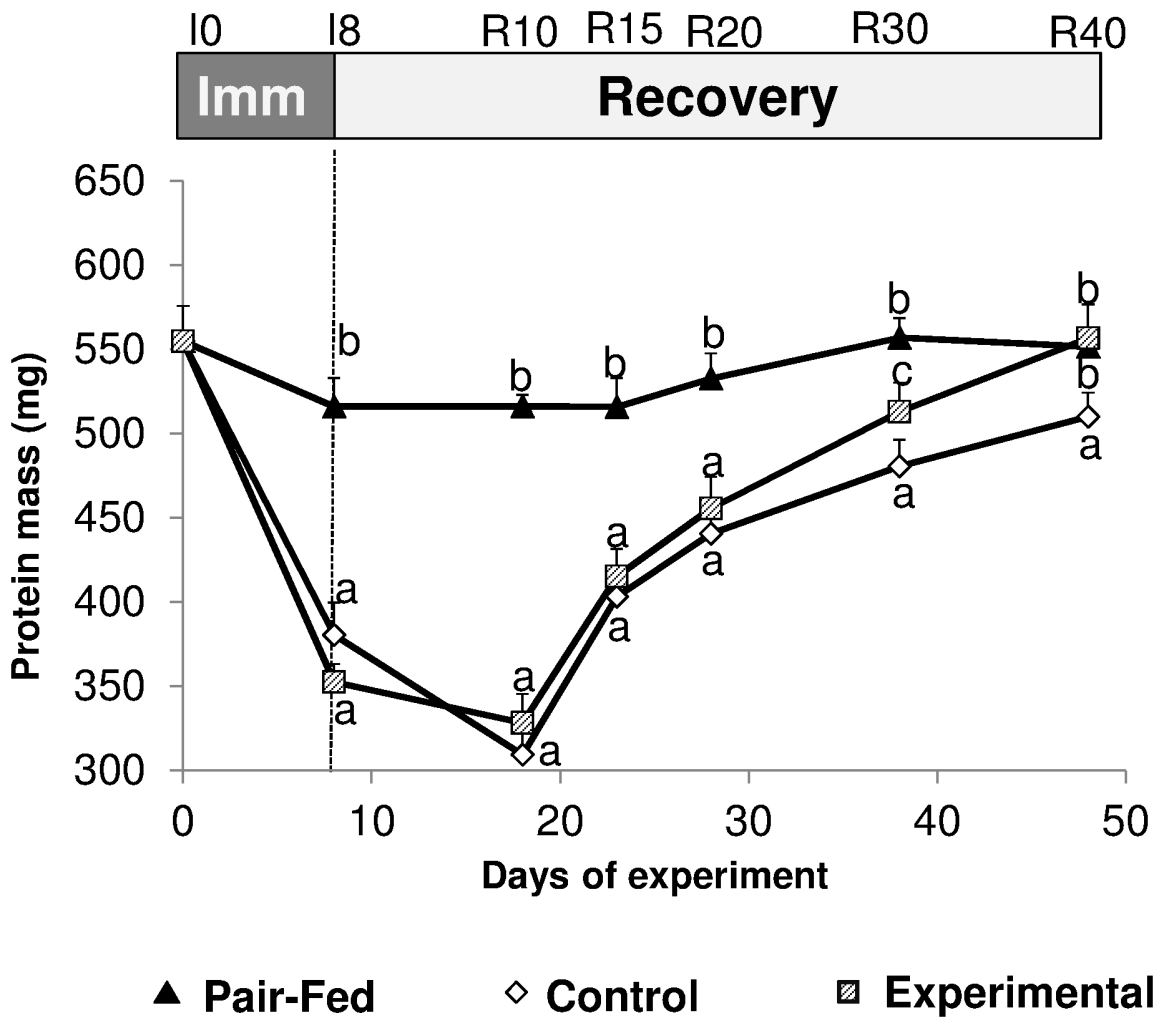
Gastrocnemius muscle total protein mass (mg) of the immobilized leg for casted control and casted supplemented rats and of one of the legs of the non-casted pair fed rats. Values are median ± SE-Median. a,b,c significantly different (non-casted Pair-fed vs casted Control vs casted AOX± Leu) (P<0.05) within each time point.

Gastrocnemius muscle protein content was reduced by 37.1% and 36.7% between I0 and I8 in CONTROL and EXPERIMENTAL animals respectively (P<0.001, I8 vs I0) (Figure 5). A small reduction in Gastrocnemius protein mass was also observed in pair fed animals (-8.6%, P<0.05, I8 vs I0, Figure 5). A diet effect was observed during the recovery period with a more rapid recovery of the gastrocnemius muscle protein mass in the EXPERIMENTAL animals vs CONTROL: protein mass was not different in EXPERIMENTAL animals vs pair fed but significantly different between EXPERIMENTAL vs CONTROL animals at R40 (P<0.05, Figure 5). 

### Effect of immobilization and recovery on protein synthesis in the post prandial and post absorptive states

#### Immobilization

**Figure 6:**
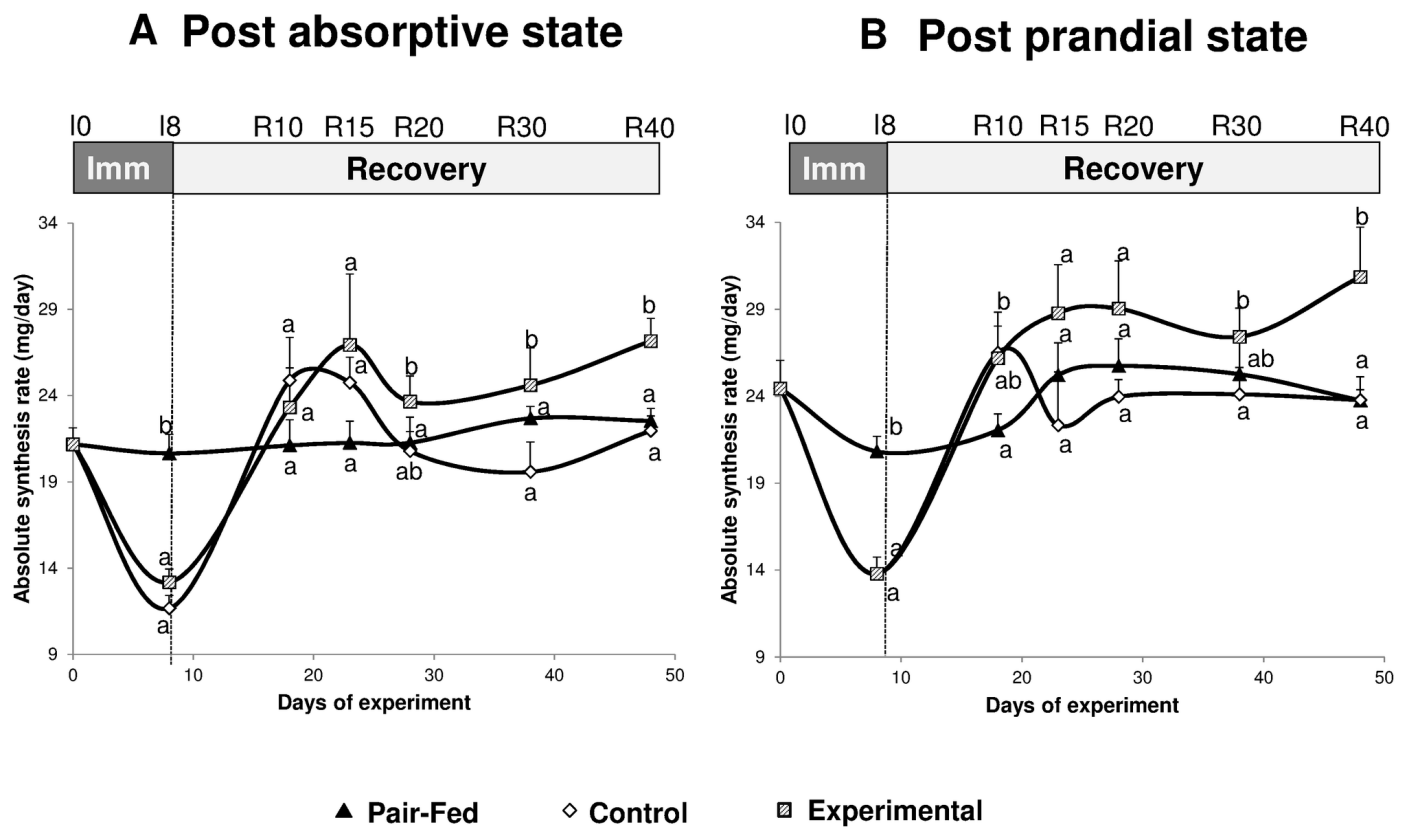
Muscle protein absolute synthesis rate (mg proteins synthesized/day) in the immobilized gastrocnemius muscle of control casted and supplemented casted rats and in the gastrocmemius muscle of pair fed non-casted rats. Protein synthesis was measured both in the post absorptive (A) and in the post prandial (B) states. Values are median ± SE-Median. a,b significantly different (non-casted Pair-fed vs casted Control vs casted AOX± Leu) (P<0.05) within each time point.

In the post absorptive state, whereas absolute synthesis rate (ASR) remained stable throughout the entire experimental period in the pair fed animals, immobilization led to a similar decreased ASR in gastrocnemius muscle whatever the diet considered (-48.7% and -39.1% between I0 and I8 for CONTROL and EXPERIMENTAL animals respectively, P<0.001) (Figure 6A). 

Similarly, in the post prandial state, a significant decreased ASR was observed in immobilized leg in both diet groups (-42.1% and -45.8% between I0 and I8 for CONTROL and EXPERIMENTAL animals respectively, P<0.001) (Figure 6B). ASR was also decreased between I0 and I8 (-16.6%, P<0.05) (Figure 6B) in the pair fed group. No significant difference of ASR in gastrocnemius was observed between CONTROL and EXPERIMENTAL animals at I8. 

#### Recovery

In the post absorptive state, ASR in the gastrocnemius muscle was similar between the three groups at R10. From R15 to R40, ASR in gastrocnemius of EXPERIMENTAL animals was above the values observed for the CONTROL group and pair fed group. This difference becomes significant at R40 (23.0% and 17.5% for EXPERIMENTAL vs CONTROL and pair fed respectively, P<0.05) (Figure 6A).

In the post prandial state, a similar trend was observed with an ASR in gastrocnemius of EXPERIMENTAL animals above the values observed for the CONTROL group and pair fed group. At R10, ASR of proteins in gastrocnemius muscle of EXPERIMETNAL group was significantly above the pair fed group (+24.6%, P<0.05) (Figure 6B). The effect was also present at R40 with a 31.3% and 28.3% increased ASR in EXPERIMENTAL animals vs CONTROL and pair fed groups respectively (P<0.05) (Figure 6B).

### Effect of immobilization and recovery on trypsin-like and chymotrypsin-like activities

Muscle protein mass is controlled by the balance between rates of protein synthesis and breakdown. The proteasome system (UPS) is involved in the breakdown of the major contractile proteins in catabolic conditions, and we previously reported that the activation of the UPS pending immobilization was normalized 20 days after cast removal [7]. Thus, the regulation of the UPS was assessed during immobilization (I8) and recovery until R20 by measurements of the main peptidase activities of the proteasomes, i.e. the chymotrypsin (CT)- and the trypsin (T)-like. 

The CT- and T-like activities of the proteasome were similar in muscles from animals either in the post absorptive or the post prandial state in all groups during the whole kinetic studied (data not shown). Thus, data obtained in both the post absorptive and the post prandial state were pooled and analysed regarding the effect of the diet.

**Figure 7:**
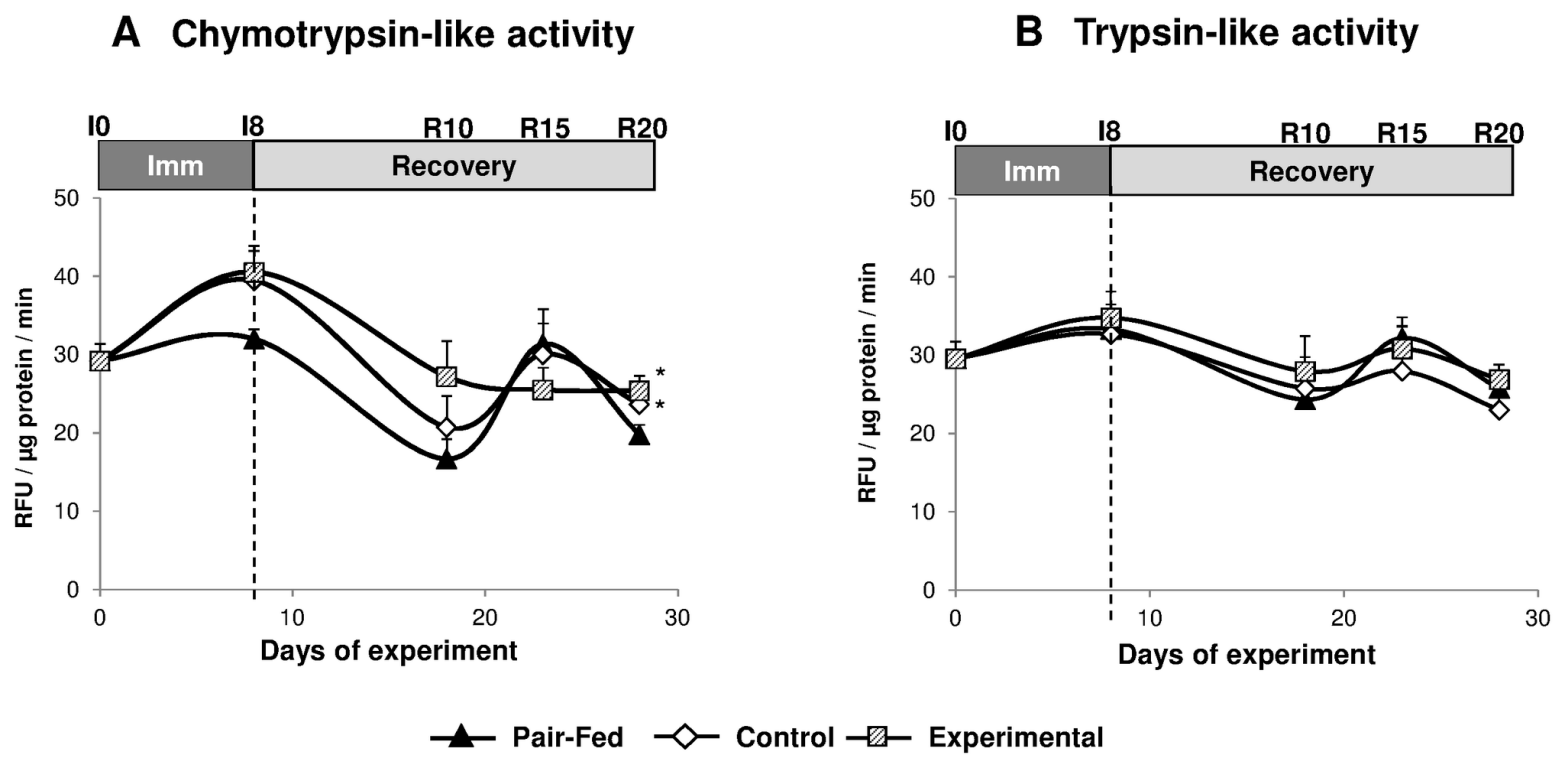
Peptidase activities of the proteasome in the immobilized gastrocnemius muscle of control casted and supplemented casted rats and in the gastrocmemius muscle of pair fed non-casted rats. The chymotrypsin-like (A) and the trypsin-like activities (B) of the proteasome were measured using a fluorogenic substrate on partially purified proteasome extracts. Data are expressed as Relative Fluorescent Unit (RFU)/µg protein/min. Values are median ± SE-Median. *significantly different (non-casted Pair-fed vs casted Control vs casted AOX± Leu) (P<0.05) within each time point.

The CT- and the T-like activities of the proteasomes did not change significantly in immobilized gastrocnemius of EXPERIMENTAL or CONTROL animals compared to the pair fed group (Figure 7). Only the CT-like activity of the proteasome increased at I8 in immobilized gastrocnemius of EXPERIMENTAL rats compared to the I0 group (+34.7%, P<0.05, Figure 7A) and at R20 in remobilized gastrocnemius of EXPERIMENTAL and CONTROL animals compared to the pair fed group (+20.5 and 29.5%, respectively, P<0.05, Figure 7A). 

### Oxidative and inflammatory markers in the gastrocnemius muscle

**Figure 8:**
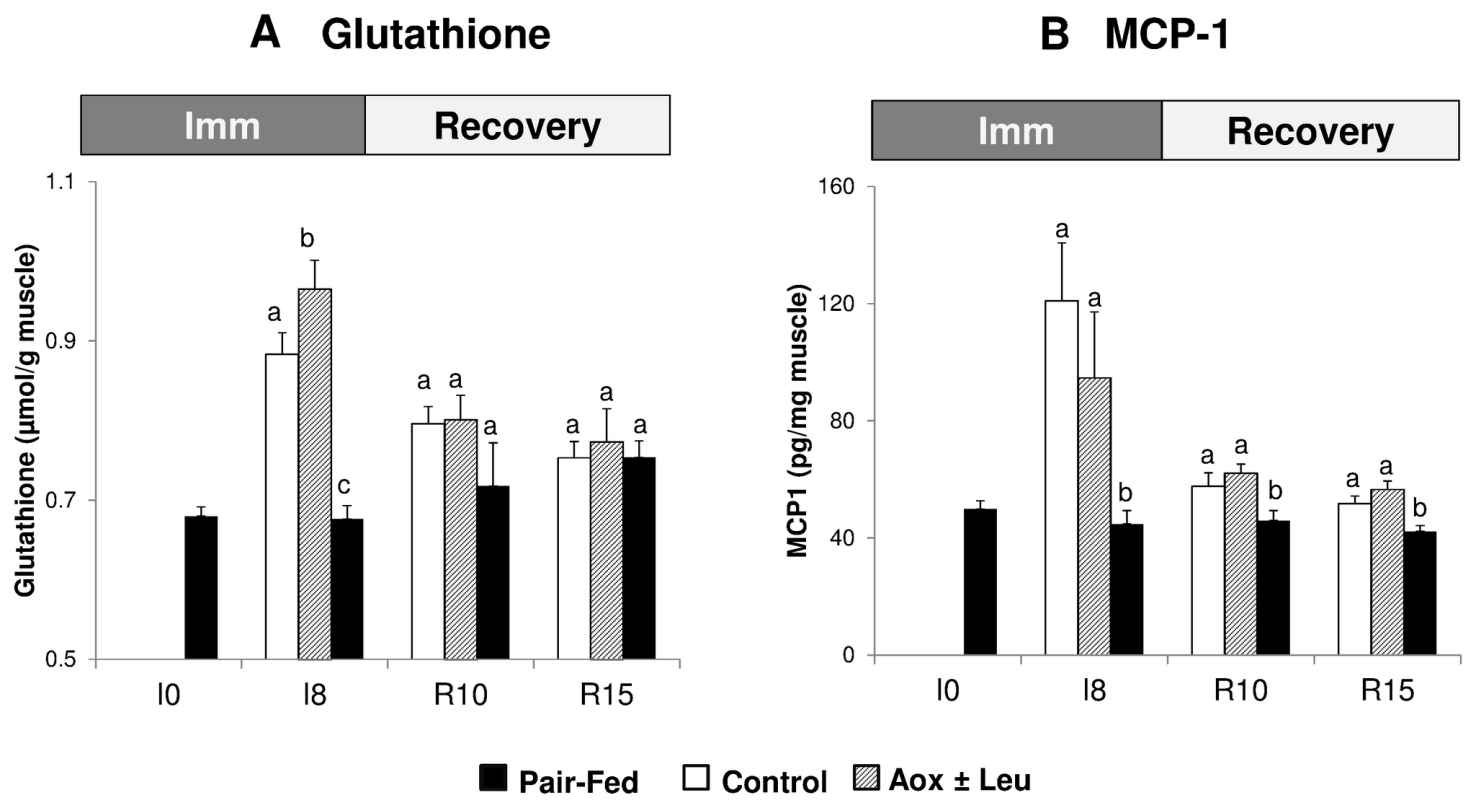
Glutathione (µmol/g muscle) (A) and Monocyte chemotactic protein-1 (MCP-1) (B) (pg/mg muscle) in the immobilized gastrocnemius muscle of control casted and supplemented casted rats and in the gastrocmemius muscle of pair fed non-casted rats. Values are median ± SE-Median. a,b,c significantly different (non-casted Pair-fed vs casted Control vs casted AOX± Leu) (P<0.05) within each time point.

Immobilization induced an increased glutathione level in the immobilized leg of the casted CONTROL and EXPERIMENTAL animals relatively to the pair fed non immobilized group (+26.1% and +40.8% vs pair fed at I8 for CONTROL and EXPERIMENTAL animals respectively, P<0.05, Figure 8A). In addition, the supplementation in antioxidants led to a more elevated level of glutathione in the immobilized leg of the EXPERIMENTAL animals vs CONTROL at I8 (+12.0% in EXPERIMENTAL vs CONTROL, P<0.05, Figure 8A). Glutathione concentrations in the immobilized animals decreased at R10 and R15 and the values were not significantly different between groups at those two time points. No significant impact of the immobilization or diet interventions could be observed in the controlateral leg (data not shown).

The monocyte chemotactic protein-1 (MCP-1) levels in the immobilized leg were strongly increased by 166.8% and 115.1% vs pair fed (P<0.05) in the CONTROL and EXPERIMENTAL animals respectively at I8 (Figure 8B). No differences between the two experimental diets were observed at I8 for MCP1. MCP1 content in muscles decreased in immobilized legs at R10 and R15. Although still significantly (P<0.05) above the values of pair fed animals, MCP1 values in the immobilized animals normalized progressively between immobilized animals and pair fed animals during the recovery period.

## Discussion

In the present study, we showed that a dietary supplementation of adult rats undergoing 8 days of immobilization followed by a recovery period with a mixture of antioxidants during immobilization and leucine ± antioxidants during the recovery period led to an accelerated recovery of muscle mass. This dietary effect of leucine ± antioxidants was related to a stimulation of muscle synthesis rate both in the post prandial and the post absorptive states during the recovery period. Indeed, whereas a muscle mass decrease of about 25% was observed at the end of the immobilization period with both diets, the muscle of immobilized control animals was still 10% lower relatively to the non-immobilized pair fed animals and no longer significantly different in the leucine ± antioxidants animals. The extrapolation of such type of accelerated recovery of muscle mass to various clinical situations associated with bed rest and muscle atrophy may be of primary importance in the convalescence of these patients.

This study also described the kinetics of modification of muscle protein mass and metabolism following 8 days of immobilization and during the recovery period. The kinetics and mechanisms of decrease in muscle protein mass during immobilization have been well described in various species [7,19,22,26,38,39]. However, little was known on the mechanisms stimulated during the recovery period with a few data concerning the adaptation of proteolytic and apoptotic pathways [7,25] and even less concerning protein synthesis [25,40], particularly *in vivo* in both the post prandial and the post absorptive states [40].

### Protein synthesis involvement in the muscle protein loss and recovery

Eight days of immobilization led to a drastic decreased protein mass in the Gastrocnemius muscle (about -37%). These data are consistent with those obtained with the same immobilization model in adult [7] and old [19] rats and were the consequence of an activation of the ubiquitin-proteasome pathway [7,19,24] and also, as shown in the present study, to a reduction of protein synthesis (both in the post-absorptive and the post-prandial states). In the present study, our data suggest a moderate activation of the UPS: only the chymotrypsin-like activity of the proteasome increased in immobilized leg when compared with the I0 group, but not with pair fed animals. We previously reported that the peptidase activities of the UPS may be strongly [7] or moderately [20] enhanced, or unchanged [41] from an experiment to another, probably resulting from the intensity of muscle damages induced by immobilization per se. The reduction of protein synthesis, generally measured in the fasted state, has already been observed in muscle of immobilized rats [30,42] or during bed rest in humans [1,43,44]. The fact that protein synthesis was decreased in both the post prandial and post absorptive states in the present study explained the rapid and important muscle loss observed after only 8 days of immobilization. Among the potential mechanisms responsible for the muscle loss, the decreased muscle protein synthesis in the post prandial state showed that immobilization rendered the muscle less capable to respond to anabolic stimuli such as meal intake or amino acids supplementation, as observed by [23,45,46] and the present study. A blunted response of the mTORC1 signalling pathway as well as a reduction of AA transporters numbers is a potential mechanism [45]. Conversely to the reduction of protein synthesis observed after immobilization, during the recovery we observed an activation of protein synthesis, which was probably the most important mechanisms explaining the protein gain since proteolysis inhibition was limited to the early stages of recovery. Indeed, we [7] have shown in the same animal model a normalization of the proteolytic systems activities within the first 10 days of recovery (or at the latest R15) and this could not explain the muscle protein mass gain observed throughout the entire recovery period of 40 days [7,19]. In our study, the stimulation of protein synthesis in both the post prandial and post absorptive states during the recovery period favoured the observed gain of muscle mass. 

The resistant state to anabolic stimuli occurring during the immobilization period (and the increased sensitivity during recovery) can be multifactorial. An alteration of the oxidative status within the immobilized gastrocnemius can be hypothesized in the present study since the glutathione content increased in the muscle at the end of the immobilization period. The increased ROS in disuse may be implicated directly in the stimulation of proteolysis and cell death via a modulation of the MAPK pathway and an activation of the NFkB pathway, as reviewed by [14]. To this direct effect, the presence of an oxidative stress during immobilization (generation of ROS) [16,19,47] has also recently been shown to be one of the determinants of the presence of an insulin resistant state of glucose transport at the muscle level (via a loss of IRS-1, p38 MAPK activation and PI-3K pathway activation) [48-50]. Both mechanisms of action of ROS could explain at least partially the muscle loss observed during immobilization by both acting on proteolytic systems (activation) and the protein synthesis response (decreased) to the amino acids supplementation in the meal [51]. 

Immobilization is also characterized by the presence of an inflammatory state, particularly at the muscle level, as can also be hypothesized in the present study with a significant increased concentration of MCP-1 content in the muscle after immobilization relatively to the non-immobilized pair fed animals. Similar data were obtained by [19,52] and were closely related to the presence of a resistant state to anabolic stimuli such as insulin [21]. In addition, a differential response of genes involved in inflammatory response and oxidative stress to insulin has been recently demonstrated in young men following bed rest [53]. 

The progressive normalization of the oxidative stress and inflammatory status during the recovery period, as shown in the present study with a normalization of the glutathione muscle concentrations 15 days after cast removal, and MCP-1 normalization within 10 days can be some factors involved in the improved muscle response to food intake during the recovery period. It is probable that these normalization processes were associated with the increased sensitivity of protein metabolism to the effect of meal.

### Impact of the supplemented diet on muscle protein loss during immobilization

 The supplemented diet did not help to prevent the muscle mass loss during the immobilization period (muscle protein and muscle mass losses were similar in both control and supplemented immobilized leg), showing that the antioxidants supplementation alone was not capable to limit the decrease in protein synthesis both in the post prandial and post absorptive states. The preservation of some components involved in the muscle antioxidant status in the supplemented animals (i.e glutathione in the present study) was not sufficient to preserve muscle mass. Although the oxidative stress has been shown to be potentially involved in the decreased sensitivity of muscle metabolism to anabolic stimuli such as insulin or AA (as detailed above), conflicting results emerge on the impact of antioxidants supplementation on immobilized muscle during the immobilization period. Antioxidants supplementation was proven inefficient (mixture of antioxidants [54]; curcumin [20]; vitamin E [55]; resveratrol [56]) or capable (vitamin E [57,58], resveratrol [59]) to counteract the catabolic impact of immobilization on muscle mass. In our study, the antioxidants supplementation was not sufficient (in terms of dose or antioxidants used) to enhance the muscle response to anabolic stimuli (such as insulin or AA). Similarly, immobilization is also characterized by the presence of an inflammatory state, as confirmed in the present study with a significant increased concentration of MCP-1 content in the muscle after immobilization relatively to the non-immobilized pair fed animals. Similar data were obtained by [19,52] and have been closely related to the presence of a resistant state to anabolic stimuli such as insulin [21]. In the present study, however, the two experimental diets did not impact differently on MCP-1 content in the muscle at the end of the immobilization period, suggesting that the antioxidants supplementation did not impact significantly on the muscle inflammation status in our rats.

### Nutritional effect during the recovery period

Data concerning the nutritional supplementations potentially capable to limit muscle mass loss during bed rest are increasing in the literature as summarized by [60-62]. However, very few studies have tackled the crucial issue of the recovery period [24,61]. In our study, after cast removal, the supplemented diet sped up the muscle mass recovery. This was explained by an increased protein synthesis rate in gastrocnemius of supplemented immobilized animals above the values obtained in the control immobilized animals both in the post prandial and the post absorptive states. This improved stimulation of muscle protein synthesis could explain the strong impact of the supplementation on the more rapid muscle protein mass recovery. Changes in UPS proteolysis, as assessed by measurement of peptidase activities of the proteasome, did not seem to be involved in the acceleration in the muscle mass recovery in supplemented animals. However, a positive impact of the supplementation on the UPS proteolytic system cannot be excluded. In fact, in this pathway the substrate is first targeted by a poly-Ub chain, and the targeted protein is then recognized and degraded by the 26S proteasome [63]. In addition, we previously reported that the UPS proteolysis was normalized with the first 15 days of recovery [7,19,20]. Thus, a beneficial effect of the supplementation may have improved UPS alterations at the polyubiquitination level and may have occurred mainly during this first phase of the recovery period. Finally, a beneficial effect of leucine supplementation may prevail also on other proteolytic systems, in particular the lysosomal pathway. In fact, autophagy, which directed substrates to the lysosome, is also important in the control of muscle mass [64] and is regulated by leucine [65-67]. For example, leucine deprivation increased autophagy in C2C12 muscle cells [65] and, conversely, elevation of leucine intake in humans decreased autophagy in muscle biopsies[67]. 

The supplementation during the recovery period consisted in a mixture of antioxidants/polyphenols and leucine for the first 15 days of recovery and in leucine alone for the later recovery period (from R15 to R40). Because antioxidants were supplemented simultaneously with leucine at the beginning of the recovery period, the beneficial effect of the nutritional supplementation during that period could be attributed to the supplementation in antioxidants mixture, leucine or the combination of both. It could be observed in the data from [19] and [47] on proteolytic/apoptotic activities and indexes of muscle oxidation response that both parameters were restored in a similar manner during the recovery period. In addition, curcumin supplementation in the diet of rats has been proven efficient during the early phases of recovery by reducing the increased Smac/DIABLO protein levels induced by immobilization [20]. 

While the action of antioxidant/polyphenols on muscle mass recovery remains to be more specifically studied, there is no doubt on the fact that leucine supplementation had a specific impact on muscle mass recovery in our study. The specific role of leucine (relatively to the antioxidants supplied during the first 15 days of recovery) was clear since protein synthesis rates were still more elevated in the muscle of the supplemented group relatively to control at R40 (whereas antioxidant supplementation was stopped at R15). Very few studies have actually tackled the concept of AA supplementation during the recovery period after bed rest and to our knowledge, only [25,68,69] have shown an anabolic or small impact of essential AA or branched chain AA on nitrogen retention or muscle mass during the recovery period after immobilization. Our study demonstrated for the first time an anabolic impact of leucine supplementation on muscle mass of adult animals during the recovery period via an activation of protein synthesis both in the post prandial and post absorptive states. The beneficial role of AA in the stimulation of muscle mass anabolism has been studied in detail [70-73] with an activation of the muscle anabolism post-prandially [74]. Among AA, leucine is known to play a major role in the stimulation of protein synthesis [75,76] and inhibition of proteolysis [77] in normal and catabolic states. During immobilization, the anabolic resistance of muscle protein metabolism to anabolic stimuli such as AA shown by [23] can be counteracted by leucine alone, BCAA or protein supplementation [26,78] but not always [68,79]. The response of protein anabolism to the AA supplementation during bed-rest depends tightly on the level of resistance of the metabolism induced by immobilization, which explains the apparent discrepancy in the anabolic potential of AA supplementation during immobilization in various studies. The originality of our results lies in the fact that AA supplementation (leucine in our case) is done when the sensitivity of protein metabolism to AA was increased (i.e., just after the immobilization period and not during the immobilization period per se). Such a progressive restoration of protein synthesis sensitivity to leucine was shown during the recovery period in other catabolic situations as glucocorticoids [28] and considering the anabolic effect of leucine supplementation on protein synthesis rates in our model, a similar mechanism of restoration of sensitivity of protein metabolism to leucine can be hypothesized.

 To summarize, we demonstrated in this study that a dietary supplementation of antioxidants/polyphenols and leucine can accelerate the recovery of muscle mass in casted adult rats. This quicker recovery was explained by an increased activation of protein synthesis rates both in the post prandial and the post absorptive states over the entire recovery period in the supplemented animals. Such nutritional strategies can hence be valuable alternative/additional means to favour recovery after disuse for patients unable to perform physical exercise during recovery in adults. More generally, there are a number of clinical pathologies associated with dramatic muscle atrophy and catabolic state. The reasons for this atrophy are from various origins but bed rest is partially responsible for this condition. Thus, the benefit of antioxidant/polyphenols and leucine supplementation observed in the present study could open new nutritional therapies for recovery period of such catabolic patients. The next step will be to validate the utilization of this combination of antioxidants and leucine in humans during and after bed rest.
